# Scrub Typhus Presenting as Unilateral Abducens Nerve Palsy: A Case Report

**DOI:** 10.31729/jnma.7275

**Published:** 2022-06-30

**Authors:** Bardan Ghimire, Rajan Bhandari, Khusraj Dewan

**Affiliations:** 1 Department of Internal Medicine, College of Medical Sciences, Bharatpur, Chitwan, Nepal; 2 Department of Gastroenterology, College of Medical Sciences, Bharatpur, Chitwan, Nepal

**Keywords:** *abducens nerve palsy*, *case report*, *Orientia tsutsugamushi*, *scrub typhus*, *zoonoses*

## Abstract

Abducens nerve palsies associated with infectious diseases are rare. Scrub typhus is an acute, febrile, infectious illness caused by *Orientia tsutsugamushi* carried out by vector mite zoonosis and is highly endemic in the so-called "tsutsugamushi triangle". The organism has been reported to be capable of entering the nervous system, causing meningitis and focal neurologic abnormalities. We report a 23 years old previously healthy girl who presented with fever, pain abdomen, vomiting and classical pathognomic black eschar mark on the right proximal medial calf region. After exclusion of other common infectious causes, scrub typhus serology immunoglobulin M was positive and was diagnosed with scrub typhus associated with unilateral abducens nerve palsy which responded to doxycycline therapy. On the background of strong clinical suspicion, we underline its significance in the interpretation of the serologic testing and its role in guiding the further treatment respectively.

## INTRODUCTION

Scrub typhus is an acute, febrile, infectious, vector mite zoonotic illness caused by Orientia tsutsugamushi mainly prevalent in the Asia Pacific rim.^1^ It is transmitted to humans through the painless bite of a larval trombiculid mite (chigger), occurring on any part of the body to acquire a blackened crust forming an eschar.^2^ Organisms disseminate after initial inoculation into the skin with presenting clinical features mainly of fever, headache, myalgias and severity ranging up to multiorgan failure and even death.^3^ Most documented neurological manifestations are encephalitis, transverse myelitis, cranial nerve abnormalities, meningitis and polyneuropathy. Abducens nerve palsy has rarely been documented.^4-8^

## CASE REPORT

**Figure 1:**
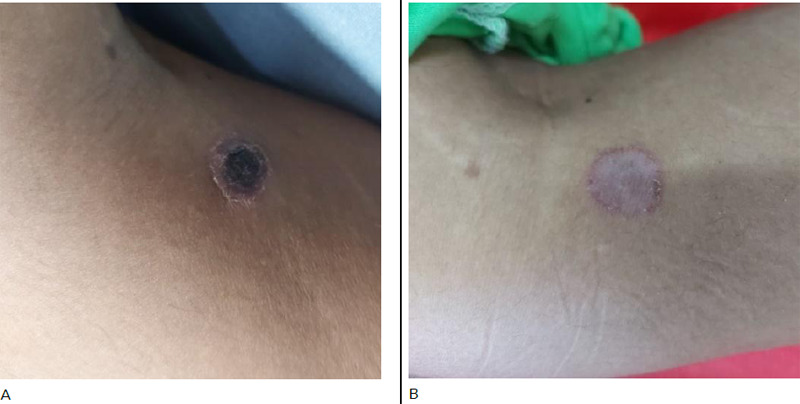
A) Black eschar was noted on the right proximal medial calf region at the time of admission, and B) After treatment, the eschar healed with residual hyperemia.

A 23 years old previously healthy female, a farmer and cattle rearer by occupation presented to the emergency room with fever, generalized abdominal pain and vomiting for 4 days. The patient was icteric and febrile, the temperature noted was 38.44°C. The abdomen was tender to palpate, and more localized on the right hypochondrium without rigidity or guarding. An eschar was noted in the right proximal medial calf region (Figure 1).

**Figure 2:**
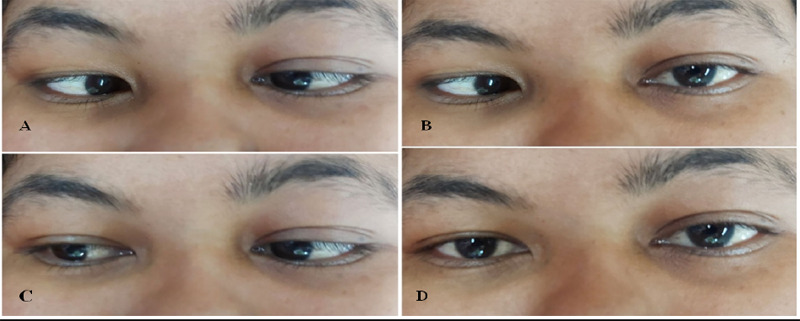
A), B) Esotropia in primary position, limited abduction in right eye on 3^rd^ day of admission, and C), D) No esotropia in primary position and no limitation of abduction in right eye after 5^th^ day of treatment with doxycycline.

Blood tests showed leukocytosis (24,380 cells/cumm) and thrombocytopenia (101,000 cells/cumm) with normal haemoglobin level (12.8 mg/dl). Liver function tests showed increased aspartate aminotransferase (355.9 IU/l), alanine aminotransferase (208.9 IU/l), alkaline phosphatase (305 IU/l), gamma-glutamyltransferase (202 IU/l) and total bilirubin level (3.0 mg/dl) with conjugated and unconjugated bilirubin noted as 1.9 mg/dl and 1.1 mg /dl respectively. Prothrombin Time/International Normalized Ratio was noted as 16.2 seconds/1.39 respectively. Amylase (32 IU/l) and lipase (22 IU/l) were normal. Tropical serology reports (malaria/leptospira/dengue/scrub typhus) were negative. Renal Function Test (RFT) was normal. Blood and urine culture reports were sterile. High-resolution Computed Tomography (HRCT) of the chest was normal. Ultrasonogram (USG) of abdomen and pelvis revealed mild hepatosplenomegaly. Contrast-enhanced Computed Tomography (CECT) of the abdomen and pelvis also revealed hepatosplenomegaly with minimal ascites. The patient was admitted to the Intensive Care Unit (ICU) and managed with intravenous antibiotics and intravenous fluids. On the third day, pain in the abdomen, vomiting and fever subsided but she complained of a sudden onset of diplopia and blurring of vision (Figure 2).

She was consulted with an ophthalmologist and her ocular motility test revealed right-sided lateral rectus palsy. The pupils were isochoric. The fundal examination was normal. Facial sensation and motor function were normal. Ptosis or diurnal variation of the diplopia was not observed. There was no nystagmus or any cerebellar dysfunction and no other abnormal neurologic findings were seen. Magnetic Resonance Imaging (MRI) brain was done which revealed a normal scan. The patient was febrile again on the 5^th^ day of admission with a maximum temperature noted of 38.5°C. Considering the clinical history, examination and relevant investigations, common tropical infections, acalculous cholecystitis, and Space-occupying Lesions (SOLs) in Central Nervous System (CNS) and cerebrovascular diseases were ruled out. Scrub typhus serology (IgM) was repeated which came out to be IgM positive. The patient was initiated on doxycycline (Intravenous 100 mg BD). Fever, myalgia and diplopia gradually improved after five days of treatment with doxycycline. The eschar also healed with residual hyperemia. Laboratory abnormalities were also resolved. On follow up, the patient was fine and doing well with no new complaints.

## DISCUSSION

Scrub typhus is a mite borne acute infectious disease caused by *Orientia tsutsugamushi* where humans are accidental hosts and are highly endemic in the so-called “tsutsugamushi triangle".^9^ Only five cases of scrub typhus associated with abducens nerve palsy have been reported.^4-8^ Age, history, clinical findings and neuroimaging (preferably MRI) decide the differential diagnoses and work-up of patients with an abducens nerve palsy. Our patient was non-diabetic, non-hypertensive, without any vascular risk factors and had no history of paresis. Infection as the cause of abducens nerve palsy is well known. Abducens nerve can be affected along its subarachnoid course, specifically, as it ascends the clivus and turns 90 degrees anteriorly to enter Dorello's canal. The nerve has a longer course. Indeed, lesions located elsewhere intracranially can secondarily affect the abducens nerve (an example of the poor localizing value of a 6^th^ nerve palsy).^10^ There are at least three pathogenetic mechanisms for the development of abducens nerve palsy in scrub typhus. First, by vasculitic/perivasculitis infarction of the vasa-nervorum of the sixth cranial nerve, second, by intermittent temporary/persistent compression with exudates percolating through the subarachnoid space around the nerve resulting in focal demyelination/ischemia. Finally, by stretching the points of fixation and areas of attachment to the base of the skull. Similar findings with other studies include: young patient; female sex; eschar was present in four out of five cases; lateral rectus palsy of the right eye and the abducens nerve palsy resolved in all the cases.^4-8^

Several methods are effective for the diagnosis of scrub typhus including Enzyme-linked Immunosorbent Assay (ELISA), Immunoflorescence Assay (IFA), Immunochromatographic Test (ICT), Weil-Felix, Polymerase Chain Reaction (PCR) and Loop-mediated Isothermal Amplification (LAMP). Other immuno-based methods like IFA and ELISA are most outranked for the detection of scrub typhus due to their higher sensitivity and specificity, but not vigorous to lay bare the infection at early stages and need the convalescent sampling for verification of positive samples.

On another deed, PCR based methods becoming acceptable over the era due to their dexterity in early-stage diagnosis with higher specificity and sensitivity but lack its easy availability and applicability in circumstances of scrub typhus due to the variegated genetic makeup of *Orientia tsutsugamushi* among its serotypes. In the first week of illness, the rapid diagnostic IgM ELISA may provide false negative results as noted in our patient, which is an injudicious interpretation of the serologic test. This delays the diagnosis, specific treatment and recovery.^11^

To conclude, this report arguably describes scrub typhus with abducens nerve palsy with an “eschar", which improved with doxycycline within 5 days without sequelae. Our case had significant improvement to full recovery at the time of discharge. Clinicians need to consider scrub typhus in the list of differential diagnoses of any sudden onset focal neurological deficit with clinical, laboratory or imaging evidence of infection in an endemic area. Delay in diagnosis may lead to multi-organ-dysfunction syndrome and death, in this, otherwise curable disease. Provided that clinical background and serologic tests are wisely taken into account together, rapid ELISA tests are rapid, cost-effective and highly sensitive screening tests in low-resource settings.
